# Work resumption at the price of distrust: a qualitative study on return to work legislation in the Netherlands

**DOI:** 10.1186/1471-2458-13-153

**Published:** 2013-02-19

**Authors:** Nicole Hoefsmit, Angelique de Rijk, Inge Houkes

**Affiliations:** 1 Department of Social Medicine, Research school Caphri, Maastricht University, P.O. Box 616, Maastricht, 6200 MD, the Netherlands

**Keywords:** Cooperation, Employee, Employer, Legislation, RDIC model, Return-to-work, Sick leave

## Abstract

**Background:**

Return to work (RTW) after sick leave is considered necessary to support the employees’ health. Cooperation between employees and employers may encourage employees’ RTW, but is hampered by bottlenecks that we do not completely understand. Dutch legislation means to support this cooperation and allows trying RTW during two years. The Resource Dependence Institutional Cooperation (RDIC) model has been developed for studying cooperation in public health. Study aims were to get insight into the degree of cooperation between Dutch sick-listed employees and employers, how this (lack of) cooperation can be understood, and how valid the RDIC model is for understanding this (lack of) cooperation.

**Methods:**

This qualitative study was based on in-depth interviews with 8 employees and 8 employers. Employees reported sick for 1.5-20 months for various reasons. Interviews were analysed using an interpretative approach and pattern matching.

**Results:**

Cooperation was lacking early during sick leave. Later on there were regular meetings, but employers decided about RTW without consulting the employees. Particularly employers were motivated to cooperate during the first year, while employees were especially motivated during the second. This could be understood by experienced dependence; employees (first year) and employers (second year) did not consider cooperation to be important for achieving medical recovery (employees) or RTW (employers). These divergent goals may be understood by personal norms about the timing of medical recovery and RTW. Legislation was particularly effective regarding employer behaviour in year 1 and employee behaviour in year 2. Employees distrusted their employers during the first year, while employers reported to distrust the employees during the second year. Besides, employees and employers experienced a moderate ability to cooperate. This could be understood particularly by having moderate knowledge about legislation. The RDIC model appeared to be valid to understand the cases studied, but the additional factor distrust also played a role.

**Conclusions:**

Legislation appeared to support cooperation, but awareness of a mutual dependence, trust, knowledge about the legislation and personal norms regarding recovery and RTW are also important. Professionals such as occupational physicians should support this to attain a degree of cooperation that is necessary for effective RTW.

## Background

Effective return to work (RTW) after sick leave is an important public health topic. Across the European Union (EU), early and effective RTW is considered necessary to support sick-listed employees’ health and well-being. RTW also prevents them from losing their jobs, which has adverse effects. For employers and governments, the financial benefits of paying less for sick leave and state subsidies play a role as well [1]. This fits in with the general aim of improving the sustainable employment and labour participation of all citizens [2]. Especially in view of an ageing population and the current economic crisis, sustainable employment is important. Therefore, several EU countries have put RTW after sick leave high on the political agenda.

Effective cooperation between sick-listed employees and their employers is considered a key element in early and effective RTW [3-8]. The employee’s perspective is important to understand RTW practice [9]. Many studies, however, demonstrate bottlenecks that inhibit cooperation in RTW, such as lack of communication and conflicting stakeholder opinions (see, for example, [10-15]).

Interestingly, the Netherlands seem to have the most advanced legislation of all EU countries regarding cooperation in relation to RTW. The Dutch Improved Gatekeeper Act imposes on sick-listed employees and their employers the responsibility to cooperate for achieving early and effective RTW. This legislation describes obligatory procedures for employees and employers to follow, such as composing an action plan for RTW. Occupational physicians (OPs) contracted by employers analyse the employees’ functional limitations and advise about RTW possibilities. Employees are compensated by their employers for at least 70% of their income during two years of sick leave. However, there are differences in legislation for the early and later periods of absence. Most employers pay up to 100% of the regular wage during six to twelve months, which means that the financial incentive to return to work is only truly felt after this period. If still sick-listed after two years, employees can apply for a long-term disability benefit. They will receive a pension if their earning capacity (the maximum income the person can still earn) has declined by 35% or more. The exact amount of these pensions therefore depends on the earning capacity left. The social insurance physician assesses the employees’ work ability and, also, the action undertaken by employee and employer to realise RTW during the two years of sick leave. Failure to cooperate as prescribed by law is sanctioned with, for instance, an additional year of wage payment obligation for the employer [16-18].

Despite this legislation, Dutch employees and employers still complain about lack of adequate cooperation [13,15]. Current evidence on what the bottlenecks constitute is limited. Better understanding of these bottlenecks is important, so that lessons can be learned and recommendations can be formulated for other countries. Public health professionals in RTW, such as OPs, professionals who develop interventions and policy makers can benefit from understanding the bottlenecks. A Canadian study by Maiwald et al. showed that those who design interventions often have a different perception of the support needed to return to work than the affected employees [19]. The Netherlands offer a unique case to study the potential impact of legislation on cooperation in RTW. It is important that RTW professionals gain better understanding of employees’ and employers’ cooperation and learn about the potential influence of legislation. We aim to perform an analysis of cooperation that is relevant for both policy makers in RTW and actors in practice such as employees, employers and OPs.

**Figure 1:**
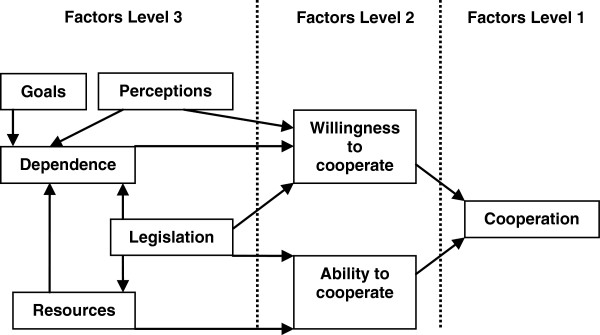
**The Resource Dependence Institutional Cooperation (RDIC) model [**[3]**].**

According to policy sciences, rules may influence cooperation and social science tells us that for example motivation can influence cooperation [20,21]. Interestingly, De Rijk, Van Raak and Van der Made developed a model which combines both perspectives. This is the Resource Dependence Institutional Cooperation (RDIC) model (see Figure 1) [3]. The theoretical model is used for understanding cooperation in public health settings such as sick leave and work resumption. It has a high internal validity as it has been constructed on the basis of established theories and was tested empirically [3].

De Rijk et al. composed the model of three levels. The first is the *cooperation* itself, defined as making agreements and acting accordingly. To understand cooperation, there is a second level in the model, which covers willingness and ability. For cooperation to exist, actors must not only be *willing* to, but also be *able* to cooperate. The model incorporates other theories in a third level, which reflects the factors that underlie the willingness and ability to cooperate. For example, actors may be unable to cooperate when they lack time to meet. More specifically, the RDIC framework covers two underlying mechanisms of willingness and ability to cooperate. The first mechanism is based on *institutions*. Institutions are rules that shape human behaviour and include legislation and sanctions. In this study, we only included legislation as an institution in the model, because we are mainly interested in the effect of the Dutch Improved Gatekeeper Law on the cooperation between sick-listed employees and their employers. An earlier study also focused primarily on the role of legislation [3]. According to the RDIC model, legislation may affect the ability to cooperate - for example by prescribing how actors may cooperate - and, also, the willingness to cooperate - for example by prescribing a minimum number of meetings [3]. The second mechanism underlying willingness and ability to cooperate was derived from *resource*-*dependence* theory [3]. This theory assumes that actors will cooperate only if they feel dependent on each other for acquiring the resources needed to achieve their own goals (such as work modifications needed for return to work) and have positive perceptions of each other. The RDIC model includes the resource-dependence theory in the factors goals, dependence, resources and perceptions [3].

In this study, we are in the first place interested in understanding the cooperation between sick-listed employees and their employers. In our analysis of this cooperation, we were inspired by the RDIC model. This raises the question whether the RDIC model is valid for understanding the cooperation. Does it help to understand why in some cases cooperation is low and in others high, or are additional factors necessary to understand cooperation in RTW? In the publication of 2007, data from a year before the Improved Gatekeeper Law were used and the dynamics might have been different [3]. We formulated the following research questions: 1) To what degree is there cooperation between Dutch sick-listed employees and employers? 2) How can this (lack of) cooperation be understood? and 3) How valid is the RDIC model for understanding this (lack of) cooperation?

## Methods

### Design and participants

For the purpose of this study, we performed semi-structured interviews and recruited a sample of three complete cases (i.e. employee and representative of employer), five single employees and five single representatives of employers. The study was not submitted to an ethical committee. According to the Dutch law (Wet Medisch-Wetenschappelijk Onderzoek met Mensen/Medical Research Involving Human Subjects Act) our type of study does not require ethical committee approval. We used purposive sampling to obtain the views of people with varying health complaints, ages, industries and places of residence (for practical reasons, we confined ourselves to the south of the Netherlands) [22].

The inclusion criteria for employees were that, at the time of the interview, they were long-term sick-listed (> 6 weeks), or had experienced long-term sick leave and had resumed work less than one year before the interview. We assumed that, after such a long period, employees would have a clear opinion about- and sufficient experience in cooperating with employers.

We studied the cooperation between absent employees on the one hand and supervisors and Human Resource (HR) professionals on the other hand. We did not interview any supervisors, because they usually have limited experience with (for example one or two) absent employees. Therefore, we chose to interview HR professionals on different levels (HR officers and HR managers) who have extensive experience with absent employees. We also interviewed a director occupational healthcare, who was involved in policy-making about and directing the organisation’s occupational health services, for example by OPs or psychologists.

We aimed to recruit as many complete cases (i.e. employee and HR professional) as possible. First, we recruited HR professionals using the telephone book on the internet. We searched for both small and medium enterprises (SMEs) and larger organisations in a variety of sectors such as construction. The phone book provided us with the contact information of the organisations. At the end of the interviews with the HR professionals (which took place at their workplaces), we asked them to give us a referral to one particular employee, which resulted in three complete cases. Some employers refused to give referrals without explicit argumentation, or did not know of an employee who would be willing to participate. In these cases, we used other strategies to recruit employees. We randomly contacted four psychologists and four physiotherapists who treat sick-listed employees through their professional associations’ websites. We asked each of these professionals to invite one client who met the inclusion criteria to participate in the study. Clients interested in participating subsequently gave their therapists permission to forward their telephone number to one of the researchers. In this way, three psychologists helped us to recruit three employees and one physiotherapist provided us with an additional employee. Finally, we recruited one additional employee through the first author’s personal network. The final sample of employees had been absent from work due to sickness between 6 weeks and 20 months. As Table 1 shows, the sample covered both male and female employees of different ages and who had diverse health complaints. We stopped recruiting new participants when saturation was achieved.

**Table 1:** Characteristics of participants

**Characteristic**	**Employee (8)**	**HR professional (8)**
Health complaints	psychological (3) / physical (2) / both (3)	
Age	<45 years (4) / ≥45 years (4)	
Gender	male (4) / female (4)	male (4) / female (4)
Absence duration	<1 year (5) / ≥1 year (3)	
Organisational size	>150 (6) / 20–150 (2)	>150 (7) / 20–150 (1)
Sector	profit (5), cases* (2) / non-profit (3), cases* (1)	profit (5), cases* (2) / non-profit (3), cases* (1)
Industry	production industry (2), healthcare (2), education (1), commercial (3)	technical (4), healthcare (2), education (1), commercial (1)
Profession	cook (1), salesman (2), process operator (2), teacher (1), administrative assistant (1), management assistant (1)	HR officer (4), HR manager (3), director occupational healthcare (1)

* case = a pair consisting of an employee and an HR professional.

### Data collection

The first author (NH) visited all participants at their homes (employees) or workplaces (HR professionals) for the semi-structured interviews. One interview lasted about 30 minutes and all others about two hours. The interview guide for both types of actors covered topics related to the cooperation between employees and the HR professionals, such as whether meetings had been arranged, the issues that were discussed and the challenges met in achieving RTW. The guide also covered contacts with other actors, such as health care professionals and the employees’ social environment (family, friends), so as to judge the influence of actors not included in the study on the employees’ and HR professionals/supervisors’ decisions regarding RTW.

Employees were asked to talk about their situation and the HR professionals about their experiences with sick-listed employees in general. The guide was used flexibly to enable participants to raise other issues they considered relevant. To challenge the accuracy and completeness of the information given, the interviewer prompted the participants using questions such as: Why…? Can you give an example? All interviews were tape-recorded with the participants’ consent [22].

### Data analysis

All interviews were transcribed verbatim. We started by reading and re-reading all transcripts to familiarise with the data. To answer our first research question (To what degree is there cooperation between Dutch sick-listed employees and employers?), we used pattern matching to compare the ‘expected cooperation’ (obligatory actions prescribed by legislation) to patterns of ‘actual cooperation’ [23]. To define the ‘expected cooperation’, we studied the text of the Dutch Improved Gatekeeper Law and practical guidelines about the law for employers and OPs [16-18]. This provided us with an overview of the official agreements on the cooperation between employees and employers. This included both the type of obligatory actions and the time when cooperation was required to take place. Then, we used the employee interviews to study ‘actual cooperation’ for each employee separately, followed by the overall degree of cooperation.

To answer our second research question (How can the (lack of) cooperation be understood?), we performed a data-driven analysis of interviews with the employees and HR professionals. We coded all interviews by searching for fragments that were consistent and meaningful parts (open coding). Then, we abstracted, defined and delineated concepts and decided about their relevance (axial coding). During the coding process, we were inspired by the RDIC model [3]. Next, we performed constant comparisons between and within cases to further refine the concepts. Then, definite themes were defined [24].

Finally, and to answer our third research question (How valid is the RDIC model for understanding this (lack of) cooperation?), we also applied pattern matching [3,23]. Pattern matching is an analytic strategy in theory-testing with cases. In this study we used pattern matching to compare the degrees of cooperation, motivation and ability that were observed in the data- to the expected ones. First, we quantified (by estimating these concepts as low, moderate or high) the actual/observed degrees of cooperation, motivation and ability in employees and employers using the interview data. Next, we compared the degree of cooperation based on motivation and ability (expected degree) with the actual degree of cooperation as observed in the data. The result of this comparison gives an indication of how well the theory covers the concept of cooperation. This comparison was also made for the concepts of motivation and ability. The expected degrees of motivation were defined using the concepts of dependence, legislation and perceptions. To define the expected degrees of ability, we used the concepts of resources and legislation. Thus, pattern matching may help to systematically analyse the fit of a model to the data [23].

Data analysis was performed manually. To ensure peer validity, the first author (NH) frequently discussed the analyses and the results thereof with the other authors (AdR, IH).

## Results

Below, we present the results regarding cooperation (research question 1). In a second paragraph we describe how this cooperation (or the lack thereof) can be understood (research question 2). In the third paragraph we present the findings regarding the validity of the RDIC model (research question 3).

### Cooperation (research question 1)

Legislation requires employees and employers to 1) meet, 2) discuss the progress of RTW, 3) share decision-making on possible RTW and 4) purchase professional RTW interventions if necessary (Table 2).

**Table 2:** Cooperation between employees and HR professionals

		**Year 1**		**Year 2**	
**Type of agreement**		**Expected**	**Observed**	**Expected**	**Observed**
	1. Meetings	+ (1,2,3,4,5,6,7,8)	+ - (1,2,3,4,5,6,7,8)	+ (1,2,3,6)	+ (1,2,3,6)
	2. Mutual exchange of information about RTW possibilities	+ (1,2,3,4,5,6,7,8)	- (1,2,3,4,5,6,7,8)	+ (1,2,3,6)	- (1,2,3,6)
	3. Shared decision-making about RTW	+ (1,2,3,4,5,6,7,8)	- (1,2,3,4,5,6,7,8)	+ (1,2,3,6)	- (1,2,3,6)
	4. Intervention to support work resumption	+ (1,2,3,4,5,6,7,8)	- (1,2,3,4,5,6,7,8)	+ (1,2,3,6)	+ (1,2,6), - (3)
**Cooperation summarised**			-		+ -

Note. The expected cooperation consisted of the obligatory actions for employees and employers that were prescribed by the legislation. Whether the employee and HR professional or supervisor acted in line with the legislative obligations (observed cooperation) was assessed for each employee individually (see the numbers in the Table). The findings regarding cooperation were summarised afterwards.

+ yes, + - partially, - no.

Table 2 illustrates that employees and HR professionals or supervisors only partially followed through on these formal agreements. This was assessed for each employee individually. The findings regarding cooperation were summarised afterwards. It appeared that the degree of cooperation differed between the first and second year of sick leave.

#### Lack of cooperation early during the first year of sick leave

Table 2 shows that there was a low degree of cooperation during the first year of sick leave. Most employees and supervisors did not meet during the first eight weeks of sick leave. One employee noted: *“There was no contact at all… He [supervisor] sent some flowers.”* (Employee4) This employee reported to be satisfied with this situation: *“I found this very pleasant.”* (Employee4) After eight weeks, most employees and supervisors met regularly. One HR professional noted that particularly in case the employee has a psychological health complaint, there is a tendency not to meet early during sick leave: *“.. supervisors think: ‘oh, the employee suffers from burnout, that will take long’, and do not keep in touch [with the employee].”* (HR professional6) Some other employees and supervisors met only once during the first year: *“My boss came to visit me once.”* (Employee2) This employee showed slight disappointment: *“..I must say that I did not hear anything from my boss in that phase [early during sick leave]..”* (Employee2)

Generally, employees and supervisors did not exchange information about possibilities for work resumption. Most employees only informed their supervisors about their medical recovery: *“I have always kept them informed.”* (Employee6) This employee noted that the supervisor did not undertake action either to exchange information: *“He [supervisor] did not make any effort to get to know my situation.”* (Employee6)

None of the employees and supervisors mutually decided about the employees’ work resumption. Employees who resumed work within one year, usually consulted their care professionals or OPs: *“My OP advised to start working again..”* (Employee4)

The organisations offered the services of an OP to their employees early during sick leave: *“We only offer an OP.”* (HR professional6) This HR professional reported not to purchase RTW interventions such as a comprehensive RTW trajectory for employees early during sick leave.

#### Supervisors use their power and decide about RTW during the second year of sick leave

The degree of cooperation was moderate during the second year (Table 2). Now, all employees and supervisors met regularly. One HR professional noted: *“You have to stay in touch with the employee..”* (HR professional6)

Similar to the first year, employees and supervisors did not exchange information about return to work possibilities later during sick leave. Supervisors used their power and decided about the employees’ RTW, without consulting the employees. These supervisors decided for their employees to either resume work immediately or the employers would dissolve their labour contracts after two years of sick leave. The supervisors based their decision on the OP’s estimation of whether the employees would be employable within the next few weeks. An HR professional noted: *“An employer does not benefit from an employee that cannot move around [is not employable].”* (HR professional1)

Further, HR professionals reported that the organisations purchased professional interventions *“to help the employee resume work at another organisation.”* (HR professional5) This, for example, was coaching to find a new job. These professional interventions were offered only in case the labour contract would be dissolved.

### Understanding cooperation or the lack thereof (research question 2)

Table 3 describes the factors related to ‘understanding cooperation’.

**Table 3:** Understanding cooperation

			**Year 1**	**Year 2**
Motivation^a^		Employee	- (1,2,3,4,5,6,7,8)	+ (1,2,3,6)
		HR professional	+ (1,2,3,4,5,6,7,8)	+ - (1,2,3,4,5,6,7,8)
Ability^a^		Employee	+ - (1,2,3,4,5,6,7,8)	+ - (1,2,3,6)
		HR professional	+ - (1,2,3,4,5,6,7,8)	+ - (1,2,3,4,5,6,7,8)
Understanding motivation to cooperate	Experienced dependence on the other ^a^	Employee	Goals: primary goal is medical recovery (1,2,3,4,5,6,7,8) / Resources: medical treatment (1,2,3,4,5,6,7,8), time without contact with employer (4,5) / Feelings of dependence on employer: - (1,2,3,4,5,6,7,8)	Goals: primary aim is RTW (1,2,3,6) / Resources: intervention to find new job (1,2,6), modified or new work (3) / Dependence: + (1,2,3,6)
		HR professional	Goals: primary aim is RTW (1,2,3,4,5,6,7,8) / Resources: information about employees’ RTW possibilities (1,2,3,4,5,6,7,8), effort from employees to achieve RTW (1,2,3,5,7) / Feelings of dependence on employee: + (1,2,3,4,5,6,7,8)	Goals: primary aim is RTW (1,2,3,4,5,6,7,8) / Resources: information about employees’ RTW possibilities (1,2,3,4,5,6,7,8) / Dependence: - (1,2,3,4,5,6,7,8)
	Perception of the other^b^	Employee	+ (1,2,5), - (3,4,6,7,8)	+ (1,2), - (3,6)
		HR professional	+ - (1,2,4,6,7,8), - (3,5)	+ - (7), - (1,2,3,4,5,6,8)
	Legislation^c^	Employee	- (1,2,3,4,5,6,7,8)	Legislative reduction pay + (1,2,3,6)
		HR professional	Feels responsible for meeting legislative requirements + (1,2,3,4,5,6,7,8)	+ (1,2,3,4,5,6,7,8)
	Distrust of the other^a^	Employee	+ (1,2,3,4,5,6,7,8)	+ - (1,2,3,6)
		HR professional	- (1,2,3,4,5,6,7,8)	+ (1,2,3,4,5,6,7,8)
	Norms about the goals^d^	Employee	+ (1,2,3,4,5,6,7,8)	+ (1,2,3,6)
		HR professional	+ (1,2,3,4,5,6,7,8)	+ (1,2,3,4,5,6,7,8)
Understanding ability to cooperate	Legislation^c^	Employee	- (1,2,3,4,5,6,7,8)	- (1,2,3,6)
		HR professional	Legislation supports planning meetings + (1,2,3,4,5,6,7,8)	- (1,2,3,4,5,6,7,8)
	Resources^e^	Employee	Time to meet + (1,2,3,4,5,6,7,8), knowledge of law + (1) / - (2,3,4,5,6,7,8) feeling well enough - (1,5,6)	Time to meet + (1,2,3,6), knowledge of decision discretion regarding RTW - (2,3,6)
		HR professional	Time, place to meet + (1,2,3,4,5,6,7,8), policy, budget + (1,2,3,4,8) / - (5,6,7), communicative skills - (1,2,3,4,5,6,7,8), knowledge about information employers are allowed to ask employees for - (1,2,3,4,5,6,7,8)	Time, place to meet + (1,2,3,4,5,6,7,8), communicative skills - (1,2,3,4,5,6,8), policy, budget + (1,2,3,4,8) / - (5,6,7)

a + high, + - moderate, - low.

b + positive, + - neutral, - negative.

c + high degree of favourability, + - moderate favourability, - low favourability.

d + norms about the timing of medical recovery and RTW are experienced.

e + has resource, - lacks resource.

It appeared that (the lack of) cooperation can be understood by A) the employees’ and supervisors’ (lack of) motivation to cooperate regarding work resumption, and B) employees and supervisors were not completely able to cooperate. Again, we see differences between the first and second year of sick leave, which were important for understanding the cooperation between employees and supervisors.

#### (Lack of) motivation to cooperate

Early during sick leave, employees reported to lack the motivation to cooperate: “.. *I did not want any contact.”* (Employee4) Supervisors, on the other hand, felt highly motivated to cooperate. An HR professional noted: *“We aim to meet an employee early during sick leave.”* (HR professional1) As opposed to this first year, the supervisors’ motivation to cooperate declined later during sick leave. Employees, however, reported that they became highly motivated to comply with the actions undertaken by supervisors. *“I am happy that my employer offered the opportunity to do temporary work.”* (Employee1)

Below, the factors are described that appear to be important for understanding the employees’ and supervisors’ motivation to cooperate: lack of experienced dependence on each other for achieving medical recovery (employee) or RTW (HR professional and supervisor), positive and negative mutual perceptions, legislation stimulates particularly the supervisor (first year) or the employee (second year), distrust and norms about the employees’ and HR professionals’ goals (Table 3).

##### Lack of experienced dependence on each other for achieving medical recovery (employee) or RTW (HR professional and supervisor)

Employees primarily focused on medical recovery during the first year of sick leave. The focus of cooperation shifted towards RTW during the second year of sick leave. Employees did not feel dependent on the employers for achieving medical recovery early during sick leave, while HR professionals and supervisors tended not to feel dependent on the employees for achieving RTW later during sick leave.

##### Year 1: employees focus strongly on medical recovery and health care professionals

Employees primarily focused on their medical recovery early during sick leave: *“I find it very important to improve my condition.*” (Employee1) They believed that: *“You can resume work only after finishing all the hospital-related things.”* (Employee7) As a result, employees considered medical treatment to be important for supporting their medical recovery. They focused on their health care professionals rather than their supervisors. *“I have benefited greatly from the psychologist.”* (Employee5) An employee who had psychological complaints noted the importance of social support for medical recovery as well: *“My coach provided theoretical support. My partner, daughter and friends supported me on the level of me as a person.”* (Employee4) These employees felt a tension in relation to their supervisors as they considered time without any contact with the supervisor an important condition for medical recovery. One employee explained: *“[In case of more contact with the supervisor]: ‘..I would have resumed work way too early. That would have made me even more ill than I already was.”* (Employee4)

In contrast to the employees (who primarily aimed for medical recovery), the HR professionals reported that supervisors aim primarily at their employees’ work resumption “*because they [the supervisors] [have to] keep an eye on the financial aspect.”* (HR professional1) Especially supervisors at SMEs thought that work resumption was urgent because, to them, sick leave was a considerable financial burden: “*It [employees’ sick leave] costs a lot and the work does not get done.”* (Employee2) HR professionals reported that they aimed to support the employees to resume work during their medical recovery: *“One does not have to wait [with RTW] until the employee is feeling completely healthy.”* (HR professional2) They attached much value to the efforts made by their employees to achieve RTW. HR professionals reported that another important condition for achieving RTW was having *“..an overview of the RTW possibilities.”* (HR professional3) This HR professional noted that knowledge about RTW possibilities may help to decide about actual RTW.

##### Year 1: employees control cooperation

HR professionals reported to feel a tension in relation to their employees. *“Some employees even say that they do not understand that we contact them..”* (HR professional5) HR professionals reported that supervisors experience difficulty to communicate with the employees about their RTW: *“Creating understanding of the employer’s situation.. that can be difficult.”* (HR professional1) Ultimately, the employees controlled cooperation; i.e. the supervisors and HR professionals adapted to the employees’ wish not to discuss RTW as they considered medical recovery to be a *“personal process.”* (HR professional6) This HR professional thought that one should not contact the employee unnecessarily during the medical recovery process.

##### Year 2: focus of cooperation shifts towards return to work

Compared to the first year of sick leave (during which medical recovery was the central focus), the focus of cooperation shifted to return to work later during sick leave. This shift was also paralleled by tensions between the employees and HR professionals.

Employees often finished their medical treatment later during sick leave, or had tried multiple medical treatments. HR professionals tended to feel annoyed about not achieving RTW (the employer’s primary goal): *“(about an employee) The employee received colour therapy, bereavement care, holistic therapy, about everything that exists. He received it all and kept on being sick-listed until we finally said: ‘we do not accept this any longer’.”* (HR professional8) One HR professional noted that this tension between aiming for medical recovery and aiming for RTW may result in a conflict: *“[About the most common source of a conflict in his organisation] An employee who thinks that he cannot work yet, while we think that he can.”* (HR professional3)

##### Year 2: HR professionals and supervisors control cooperation

HR professionals felt that they had more influence on the situation than employees later during sick leave: *“We tend to steer it.”* (HR professional5) HR professionals usually used their relatively powerful position to make employees to return to work: *“[About a conflict with employees] I clearly explain what I expect them to do.. than they are normally fine with that.”* (HR professional4) Other HR professionals may threaten employees: *“You risk us not paying you wages anymore..”* (HR professional6) (Dutch employers can ask the social insurance office for permission to stop wage payment if employees do not cooperate in trying to achieve RTW). Actions undertaken by HR professionals had effect, because most employees thought that their job was at risk. An HR professional noted: *“Employees think: they want to dump me.”* (HR professional1) These employees felt that they should put more effort into RTW. Consequently, RTW became the most urgent goal to them: *“I need to have an income.”* (Employee6) The employees felt dependent on their supervisors for modifying or renewing their tasks. One employee mentioned: *“Receptionist… that is a job they would never offer.”* (Employee6)

HR professionals, on the other hand, no longer felt comfortable waiting for the employees. *“I want a solution.”* (HR professional8) The HR professionals considered a professional’s opinion about the employees’ RTW possibilities to be important: *“Let the OP determine that.”* (HR professional3) In the end, the supervisors usually decided about the employees’ RTW themselves (immediate work resumption or dissolving the labour contract after two years of sick leave). One employee told that he was disappointed: *“I always enjoyed my work.”* (Employee6) One employee noted: *“I am just grateful that they pay me my wages.”* (Employee6) This employee felt that he could not influence the supervisors’ decision.

##### Positive and negative perceptions of each other

Some employees viewed their supervisors positively during the first year of sick leave. *“She is very supportive*.” (Employee1) Others were sceptical about their supervisors’ personal qualities. *“He cannot communicate.”* (Employee6) Most HR professionals did not have an outspoken positive or negative perception of their employees. However, some HR professionals considered their employees *“very passive”* (HR professional5) in realising their own RTW.

The employees’ positive or negative perceptions of their supervisors remained unchanged during the second year. Most HR professionals, however, had a negative perception of employees during the second year of sick leave. One HR professional complained about employees saying: *“It [return to work] does not quite work out.”* (HR professional8) This HR professional noted that some employees did not actively aim to return to work.

##### Legislation stimulates particularly the supervisor (first year) or the employee (second year)

Legislation also supported the employees’ and supervisors’ motivation to cooperate. Again, there were major differences between the first and second year of sick leave. Generally, legislation supported particularly the supervisor (first year) or the employee (second year). This is explained below.

##### Year 1: supervisors felt responsible for meeting legal requirements on cooperation

Both employees and HR professionals reported that they considered supervisors to be responsible for meeting the standards as set by law early during sick leave. For example, employees in general regarded supervisors responsible for contacting them or modifying their work. One employee noted: *“he [the supervisor] would be held responsible [by the social insurance office] if I could not resume work.”* (Employee2) Additionally, particularly the HR professionals told that they were afraid of being sanctioned for not meeting legal requirements. *“The social insurance office assesses the employer’s efforts in supporting RTW and imposes sanctions. I do not think employees are judged so strictly.”* (HR professional5) In fact, employees and supervisors who met each other only once did so because it was prescribed by law. *“We at least have to make an action plan for RTW.”* (HR professional5)

##### Year 2: legal reduction in pay stimulates employees to cooperate

Employees received a legally sanctioned reduction in pay and became aware that the two-year period of sick leave had almost expired: *“I started to realise that there is much at stake.”* (Employee6) This employee thought that because of sick leave, he may eventually lose his job and thus cooperation with the supervisor became more important.

##### Distrust

Some HR professionals emphasised the importance of trust in employee-supervisor relations: *“In case employees trust their supervisors, there will be more open communication during sick leave.”* (HR professional6) Another advantage of trust was noted as well: *“..both can deal easily with work-related issues [making work adaptations].”* (HR professional4) Nevertheless, during both years of sick leave, employees and supervisors experienced distrust. Distrust may have two different consequences: either avoiding meetings and exchange of information about RTW (employees, year 1) or planning the meetings that are required to abide by Dutch legislation (supervisor, year 2).

##### Year 1: Employees may distrust their supervisors

Employees experienced not to trust their supervisors (completely) early during sick leave. One employee explained: *“Of course my supervisor does not truly care about me. But that is inherent to his position.”* (Employee2) Both employees and HR professionals recognised that a violation of trust before sick leave can be the reason of sick leave: *“A conflict in the workplace can even turn into sick leave.”* (HR professional1) One employee explained: *“I told my supervisor that I had too much work. He did not agree. And there it all began.. [about the reason for sick leave].. frustration of the past years. I was so angry at my work..”* (Employee3) This employee also mentioned: *“My supervisor still does not recognise that he made a mistake.”* (Employee3) The violation of this employees’ trust before sick leave, still played a role during sick leave.

##### Year 2: HR professionals’ trust in employees may be violated because of sick leave

Later during sick leave, the HR professionals’ trust tended to be violated because of sick leave. One HR professional explained: *“..we indeed distrust an employee.. anyone can say that his general practitioner told that he should not work.”* (HR professional3) This HR professional seems to think that the employee did not do everything he could do to resume work.

An HR professional told about the consequences of a lack of trust in employees: *“Than I can be really strict..”* (HR professional5) These HR professionals planned the meetings that are required to abide by legislation. One HR professional noted: *“We are obliged to keep searching for RTW possibilities.”* (HR professional6) This HR professional mentioned to be afraid of being sanctioned for not doing enough to achieve the employees’ RTW. Thus, the legislation stimulated the HR professionals to use their power for realising cooperation regarding the employees’ RTW.

##### Norms about the employees’ and HR professionals’ goals

The employees aimed to resume work after medical recovery, while HR professionals aimed to help the employees resume work during medical recovery. These goals seem to be affected by one’s norms regarding medical recovery and work resumption. HR professionals noted that the social environment and care professionals may affect the employees’ personal norms about medical recovery and work resumption: *“The employees’ partner and friends often say: ‘stay at home’.” *(HR professional3) *“If a surgeon tells that an employee cannot work, he will not work.”* (HR professional4) The HR professionals felt stimulated by OPs or by research findings: “*Research shows that employees with psychological complaints should return to work within a couple of weeks..”* (HR professional2). This HR professional experienced a norm to aim for the employees’ work resumption during medical recovery.

#### Perhaps motivated, yet not completely able to cooperate

Employees and supervisors had a moderate degree of ability to cooperate during both years of sick leave. One HR professional noted: *“Supervisors are not entirely ‘equipped’ to support sick-listed employees.”* (HR professional4)

Several factors may help to understand the employees’ and supervisors’ moderate degree of ability, which are: time- and feeling well enough to meet, knowledge, communication skills, policies and money to support the employees’ work resumption and legislation. Generally, employees and supervisors possessed some aspects (such as time to meet) and lacked others (such as knowledge of the legislation). Below, an overview is given per aspect.

##### Time- and feeling well enough to meet

Generally, employees and supervisors experienced to have enough time to meet. One HR professional noted *“You need to make time for such meetings.”* (HR professional5) However, some employees noted that they did not feel well enough to meet their supervisors early during sick leave: *“I felt so bad, I could only stay in bed.”* (Employee5). This may have inhibited these employees to cooperate with their supervisors.

##### Knowledge

One employee reported to know the legislative prescriptions about the employees’ role in RTW and noted: *“..this may be because I work at an HR department.”* (Employee1) However, most employees told that they did not know the legislation about return to work: *“I do not know if my RTW process proceeds according to some legislation.”* (Employee4)

Generally, supervisors did not know whether they were allowed to ask employees about their medical recovery if the employees did not bring this subject up themselves (Dutch legislation does not permit employers to ask about the medical diagnosis of sick employees). One HR officer noted that in their organisation, there are supervisors who do not at all know what to do in case an employee calls in sick: *“.. [to some supervisors] I explain everything from the legislation to the agreements made with the occupational health service, step by step.”* (HR professional3) Some employees noticed that their supervisors lacked knowledge of psychological complaints: *“My supervisor keeps asking what productivity he can expect from me. He does not recognise that the course of a psychological complaint is erratic.”* (Employee 4)

In those cases in which the supervisor decided about RTW, employees did not know their decision latitude. The employees thought that: *“In the end it is the OP who decides”* (Employee2) (Dutch legislation allows employees and employers to decide on RTW, the OP has an advisory role only).

##### Communication skills of supervisors

Particularly the HR professionals spoke negatively about the supervisors’ communicative skills. HR professionals noted that supervisors find communication particularly difficult in case the employees suffered from psychological complaints: *“[about the reason why supervisors do not always succeed to discuss limitations in work-related functioning] There still is a taboo on psychological complaints.. Employees may be hesitant to talk about their situations”* (HR professional6) These supervisors’ lack of communicative skills may relate to their lack of training in supporting employees on long-term sick leave: *“..In our organisation, supervisors cannot not take part in trainings about dealing with sick leave. Only HR professionals are trained”* (HR professional4) Overall, these supervisors’ lack of communicative skills may have inhibited them to cooperate with the employees.

##### Policies and budget to support the employees’ work resumption

Some organisations lacked an extensive sick leave policy and budget to support the employees’ work resumption: *“We do not have a social worker or a psychologist.”* (HR professional6) This may have inhibited the supervisors’ possibilities to offer professional interventions. One employee noted that he was disappointed *“Some organisations pay for meditation courses or other support. They [employer] should have offered me something like that.”* (Employee8) Thereby, the organisation’s sick leave policy and budget to support absent employees may have affected the cooperation between absent employees and their supervisors.

##### Legislation supported the HR professionals and supervisors to plan meetings with employees

An HR professional explained how legislation helped him and the supervisors to plan meetings with employees: *“We hand out pamphlets [to employees] about the law [and what it says]. Most employees then understand why we have to plan meetings.”* (HR professional5) As the last quote shows, the HR professional used legislative guidelines to convince their employees of the necessity of meeting.

### The internal validity of the RDIC model (research question 3)

Table 4 describes the expected degrees of cooperation, motivation and ability (which are defined based on the underlying level in the RDIC model) and the observed degrees of cooperation, motivation and ability (reported by employees and HR professionals).

**Table 4:** The validity of the RDIC model

		**Year 1**		**Year 2**	
		**Expected**	**Observed**	**Expected**	**Observed**
Cooperation		-	-	+ -	+ -
Motivation	Employee	+ - / -	-	+ / + -	+
	HR professional	+ /+ -	+	+ -	+ -
Ability	Employee	-	+ -	-	+ -
	HR professional	+	+ -	-	+ -

Note. + high, + - moderate, - low.

As can be seen in Table 4, the expected degrees of cooperation matched with the observed degrees of cooperation regarding both years of sick leave (first year: low degree, second year: moderate degree of cooperation, see answer to research question 1). Further, in some cases, there was a full fit between the expected and experienced degrees of motivation. This fit existed in case the employee reported a negative perception of the supervisor and in case the HR professional reported no outspoken positive or negative perception of the employee during the first year of sick leave. During the second year, there was a match between the observed and expected degrees of motivation in case the employee reported a positive perception of the supervisor and in case the HR professional either did not report an outspoken perception of the employee or experienced a negative perception. Finally, there was no full fit between the expected and observed degrees of ability. The expected degree of ability was low in employees and high in HR professionals, whereas both employees and HR professionals reported a moderate degree of ability during the first year of sick leave. During the second year of sick leave, a low degree of ability was expected, while both employees and HR professionals experienced a moderate degree of ability.

Table 3 describes an additional factor that is not part of the RDIC model, which is the employees’ and HR professionals’ distrust. Finally, Table 3 describes the employees’ and HR professionals’ norms about the goals of medical recovery (employees’ primary goal) and RTW (HR professionals’ primary goal). We did not include personal norms in the version of the RDIC model that we used in this study (see Figure 1).

## Discussion

This study aimed to describe and understand cooperation between Dutch sick-listed employees and their employers (in this study, the employer was represented by the HR professional) and to analyse the internal validity of the RDIC model. We conducted in-depth interviews with three complete cases (i.e. employee and employer), five single employees and five single employers.

Overall, the results of the qualitative analyses showed that the degree of cooperation was low during the first and moderate during the second year. Particularly employers were motivated to cooperate during the first year, while employees were more motivated during the second year. This could be understood by experienced dependence; employees (first year) and employers (second year) tended not to consider their cooperation to be important for achieving medical recovery (employees) or RTW (employers). These goals may be understood by personal norms about the timing of medical recovery and work resumption. Legislation was particularly effective with the employers during the first year and the employees during the second year. Employees tended to distrust their employers during the first, while employers experienced to distrust the employees during the second year. Besides, employees and employers experienced a moderate ability to cooperate. This could be understood particularly by having moderate knowledge about the legislation.

### Discussion of the results

Our study has illustrated the importance of legislation. It may enforce a minimum cooperation between employees and employers - when all else has been tried and proven insufficient. For example, the legislation may stimulate the employers to use their power to plan meetings with the employees. Thus, as was also reported in the paper of de Rijk et al. (2007) - applied to the workers’ council- legislation can give employers their ‘teeth’ [3]. Still, the full potential of cooperation seems often not to be reached. It appears that for an adequate cooperation, employees and employers particularly need to ‘be aware of their mutual dependence’, ‘trust each other’ and ‘have knowledge about the legislation’. Moreover, the price of distrust is paid and might negatively influence the further relations between employee and employer.

#### Awareness of a mutual dependence for achieving medical recovery and RTW

Our results illustrate that early in absence, employees were motivated to cooperate with employers only if the employers allowed them to focus mainly on medical recovery (the employees’ primary goal). Later, however, employers were motivated to cooperate mainly for achieving RTW (the employers’ primary goal). Employees, and some employers may consider it impossible to achieve medical recovery and RTW simultaneously, which generates a certain competition instead of cooperation [3].

In fact, several studies published in the last decade have shown that symbiotic dependence, by means of which employees and employers help each other to achieve both medical recovery and RTW simultaneously, is most effective and beneficial (a win-win situation). If employees and employers cooperate in modifying work tasks and attaining RTW, they benefit in terms of health (the employees; see [1,25]), and early and sustainable RTW (from which the employers benefit; see [4-7,26]). 

Professionals such as OPs may make the employees and employers aware of their mutual dependence for achieving their own goals (employees: medical recovery/employers: RTW). Our analysis showed that the employees’ and employers’ goals are influenced by the norms regarding medical recovery and work resumption. Therefore, professionals need to be aware of the role of the norms held by for example employees’ family or care professional about the timing of RTW in relation to the medical recovery process. Intervention studies may also focus on the employees’ and employers’ awareness of their mutual dependence. Researchers involved in the present study will develop and test professional support (including coaching of employees and employers).

#### Trust

Distrust stimulated employers to plan the meetings that are required to abide by the legislation. At first sight, distrust may be functional, i.e. result in more meetings. However, our findings also showed that in these meetings, particularly the employers tended to pursue their self-interests. For example, the employers often decided themselves about the employees’ RTW. Some employees appeared to be disappointed about the employers’ decision. A cooperation based on mutual trust might have resulted in a more adequate work resumption. Ståhl mentions trust as a key condition for effective cooperation in work resumption [4].

To enhance trust, professionals such as OPs may stimulate employees and employers to communicate about matters of trust and support them to acknowledge each other’s role in the work resumption process. Empowerment of employees is another way to get more influence in the unequal relationship with the employer. However, this appears to be difficult and ambiguous because it may have excluding consequences for employees who cannot fulfil the expectations [27].

#### Knowledge of the legislation

The study results illustrate that employees and employers considered employers to be responsible for meeting the standards set by law, while officially they are both responsible. The employees’ and employers’ lack of knowledge of the Dutch law causes the law to be sub-optimally effective in increasing cooperation. As our study has shown, employees often waited for their employer to take action.

The above illustrates that employees and employers not only need to be motivated, but must also be able to cooperate. Professionals such as HR officers should inform the employees and supervisors about the legislation and develop local protocols accessible through the internet based on the legislation.

### Internal validity of the RDIC model

The RDIC model helped to understand cooperation between Dutch sick-listed employees and their employers. However, there was a mismatch between the expected and observed patterns of the employees’ and employers’ ability to cooperate. For example, employers who did and those who did not feel effects of the legislation experienced a similar ability to cooperate. Possibly, the employers felt the practical resources to be more important for understanding the ability to cooperate than the legislation. Further, the mutual perceptions differed among cases from positive or neutral to negative. According to the RDIC model, actors only feel dependent on each other for acquiring a resource if they perceive each other positively enough [3]. At this point, our data is not in line with the theory. The data show that despite the employees’ and employers’ negative perceptions, they may still feel dependent on each other. Also, in some cases there was a mismatch between the expected and observed degrees of motivation to cooperate. The degrees of motivation were similar in employees and employers with positive, neutral or negative mutual perceptions. Possibly, the legislation and feelings of dependence on each other for achieving ones goals are more important for understanding the motivation to cooperate than the perceptions. Distrust may also play a role. For example, our findings showed that distrust stimulated employees to avoid cooperation with their employers.

Although we focussed on the legislation, we also found evidence for norms playing a role. Strong norms regarding RTW exist [4] and thus, this seems a factor of importance.

### Study limitations

This study has limitations. First, we did not succeed in gathering a sample consisting only of complete cases (i.e. employee and employer). Some employers who participated in our study refused to give referrals to an employee. This might be because the employers did not know a person whom they expected to be willing to give an interview. It may also be related to distrust, which would support our finding that the cooperation between employees and employers is often paralleled by distrust.

No immediate supervisors have been interviewed on the employers’ side; only human resource officers and managers. Most human resource officers and managers had extensive experience with supporting employees on long-term sick leave. They were not involved in early, successful RTW. Their selective experience with work resumption may have coloured the human resource officers’ and managers’ perceptions of employees somewhat. Also, some employees interviewed had resumed work (but not more than one year ago). These employees were interviewed retrospectively. A retrospective interview may yield different results than an interview at the time of sick leave.

We interviewed three complete cases. These complete cases did not yield extra information compared to the other interviews, although one case enabled us to verify statements of the employee and employer against each other. This lack of additional information could be explained by the process of our data collection: we interviewed the employers before the employees. We asked the employers to talk about their experiences with sick-listed employees in general (and not about specific cases). At the end of the interviews with the employers, we asked for a referral to an employee. This employee was asked to talk about his/her situation.

The employees that we interviewed were not highly motivated to return to work quickly, which may be related to our inclusion criterion that employees had to be on sick leave for at least 6 weeks. We also noticed a tendency among employers to provide socially desirable answers, and some of them only gave referrals to employees whom they expected to speak positively about them. This can be considered an argument against the use of complete cases only. The lack of motivation to cooperate in RTW might thus in many cases be even more problematic than the data suggest - which would actually reinforce our results.

In this study, we applied the method of pattern matching to analyse the degree of cooperation and to study the validity of the RDIC model for understanding the cooperation between Dutch absent employees and their employers [23]. As part of this method, we quantified concepts such as cooperation using the interview data and documents about the legislation. It is difficult to quantify concepts based on qualitative data. Despite of this limitation, pattern matching supports a systematic and thorough analysis of the fit of a model to the data. Therefore, it appears to be an appropriate analytic strategy for this study.

## Conclusions

Our findings illustrate that legislation can ensure a minimum cooperation between employees and employers (only if this legislation includes rules regarding cooperation). However, legislation alone is not enough to achieve adequate cooperation between sick-listed employees and their employers. Awareness of a mutual dependence on each other for achieving their own goals (employees: medical recovery / employers: RTW), trust and enough knowledge about the legislation are important. Moreover, personal norms regarding medical recovery and RTW play a role in the employees’ and employers’ primary goals (employees: medical recovery / employers: RTW). Professionals such as OPs may support these factors to attain a degree of cooperation that is necessary to establish effective RTW.

## Abbreviations

OP: Occupational physician; RDIC model: Resource Dependence Institutional Cooperation model; RTW: Return to work; SMEs: Small and medium enterprises

## Competing interests

The authors declare that they have no competing interests.

## Authors’ contributions

AdR set up the strategy for this study and the analyses. NH conducted and transcribed the interviews, performed preliminary analyses. All authors discussed and improved the results of the preliminary analyses and were involved in writing and/or providing feedback on the manuscript. All authors read and approved the final manuscript.

## Authors’ information

NH is a PhD student at the Department of Social Medicine at Maastricht University, the Netherlands. Her area of study is work resumption after sick leave. She completed a Master of Work and Health at Maastricht University.

AdR (PhD) is associate professor at the Department of Social Medicine at Maastricht University, the Netherlands. She graduated in Work and Organisational Psychology at Nijmegen University in 1994. She received her PhD in Health Psychology at Utrecht University in 1999. Her thesis was named: “Fatigue in general–practice patients. An empirical study of fatigue in general practice and the development of the Quality-Quantity model for understanding fatigue”, which was published in five papers in international peer-reviewed journals. She works at Maastricht University since 1999 and has focused her research and education on psychological and sociological theories regarding work disability and re-integration into work. She led several research projects on these themes and contributed to theory development. Recently she chaired the revision of the Dutch guideline on cardiac rehabilitation, which now also includes directions for re-integration into work.

IH (PhD) is a health scientist and she works as an assistant professor at the department of Social Medicine, Maastricht University, the Netherlands (Faculty of Health, Medicine and Life Sciences). Her current research interests encompass various topics in the area of work and health such as burnout, fatigue among general practitioners, work life balance, cultural differences in work and health, domestic work, gender and health, vocational rehabilitation, labour participation of people with visual impairments, sustainable employment, as well as research methodology. She supervises PhD students who perform research in these areas and is (co-)author of various scientific papers in these fields. In addition, she is program coordinator of a B.Sc. program in Work & Health and is involved as a teacher in the bachelor programs Health Sciences, European Public Health and Medicine at Maastricht University.

## Pre-publication history

The pre-publication history for this paper can be accessed here:

http://www.biomedcentral.com/1471-2458/13/153/prepub
